# Caloric, Its Mechanical, Chemical, and Vital Agencies in the Phenomena of Nature

**Published:** 1846-07

**Authors:** 


### REVIEWS.

ART. III.—*Caloric, its Mechanical. Chemical, and Vital Agencies in the Phenomena of Nature.* By SAMUEL L. METCALFE, M.D., of Transylvania University. Two volumes, octavo. pp. 1100. London. 1843.

When estimated as an aggregate, by the value in detail of its *inherent qualties*, and by the peculiar nature of certain incidental circumstances which unite with those qualities in giving character to it, this cannot fail to be regarded, by competent judges, as one of the most extraordinary productions of the kind that has issued from the British press during the nineteenth century. Had we said, that has issued from *any press* during *any century*, whose history makes a part of recorded knowledge, it would not be easy to fasten on us the charge of either error or extravagance. We, at least, are compelled to think so, after a very careful examination of the subject. For, were we now called on, to indicate its *superior*, or even its *equal*, in most of the characteristic attributes here contemplated, we would not very promptly accept the invitation. And the ground of our hesitancy would be the difficulty of the task. In illustration and proof of this early commendation we shall offer, for the present, but a few remarks, reserving a full exposition of the subject to throw light on sundry topics designed for consideration in subsequent parts of this article.

As an *inquiry in physics*, we remember no instance, in which the work before us has been surpassed on sundry points in *depth* and *solidily;* and we challenge refutation, when we add, that, in the *compass and grandeur of its outline, it cannot be surpassed.* Of this latter assertion, unqualified as it *is*, and tinctured with *dogmatism* as it may possibly *be deemed*, the truth is indubitable, and the reason plain.

Not content with merely accompanying astronomers in their excursions through the heavens, from satellites to planets, from planets to comets and nebulæ, from them to suns and astral systems, and to all other *visible* bodies—not content with this range through "starry space," the *inquiry* leaves far behind it not only the Newtons, Laplaces, and Herschels themselves, but also the reach of their most powerful glasses, and terminates its career only at the boundry of *material creation.* Nor do we thus express ourselves in the language of either figure or fancy, but in that of sober and unexaggerated truth. And it need hardly be added, that, to human research, the *passage* of the boundry, to which the inquiry thus extends, is irrevocably interdicted to human research, by the limited capacity of the human intellect.

But this interdict, positive and immutable as it is, imposes on the inquirer, in his philosophical investigations, no *actual privation or restraint.* Though it bars him by an outline, beyond which conjecture itself can never penetrate; yet within that line lies a field (most of it still untrodden) containing "ample room and verge enough," and enriched by a store of the choicest objects of research, sufficiently abundant, to afford endless action and enjoyment to all the most high-gifted minds and ambitious spirits, that can ever engage in the exploration and culture of it.

Of this truth no writer is more thoroughly informed than our author; nor has any one more highly and justly prized the knowledge of it, or made more earnest and persevering efforts to employ it in the production of useful results. Nor is this all that truth permits and duty impels us to say on the subject.

A mere ambition to distinguish himself in the higher walks of physical science here referred to, is not the only aptitude to that effect which Dr. Metcalfe has manifested. Far from it. Yet it *is* an aptitude of no ordinary significancy and value; because, without *an ambition to excel*, no one will ever toil after excellence, and of course will never attain it. For it is as certainly and necessarily the product of toil, as is day-light of the presence of the solar rays, and darkness of their departure. But our author, as will hereafter more conclusively appear, has other fitnesses beside ambition. He has shown uncommon *ability* in the performance of his part, in the great enterprise of cultivating and improving the knowledge of nature, which is rapidly shedding so pre-eminent a lustre on the nineteenth century. And that ability indicates in him no less the existence of a strong, penetrating, and well disciplined intellect, than it does the possession by him of an inextinguishable thirst for knowledge, an industry in pursuit of it which never faulters, and a resolution to master it which no difficulties or privations can subdue. And such, as will be evinced, by facts to be presently adduced, are the mental characteristics he has abundantly manifested, in his laborious preparation of the work we are examining. Were we, from what we know of the history of that preparation, to attempt the composition of a motto for Dr. Metcalfe, expressive of the determination he fostered, the labors he accomplished, and the obstacles he encountered and vanquished in the course of it, we do not perceive that we could acquit ourselves better, than by recommending to his adoption the following words:—AUT VICTOR ET FAMA, AUT VICTUS ET NIHIL —for *self-sacrifice* or *success* was obviously the end he held constantly in view.

We hold it not impossible, that a few of the foregoing remarks will be regarded, by some persons, as more in the style and spirit of eulogy, than of criticism or review.— Should such be the opinion of individuals or the public; though, as far as *words* are concerned, it will be allowed to pass unnoticed; yet shall it be met and refuted by *facts*. Special references to Dr. Metcalfe's volumes will be made, and numerous passages extracted from them, fully sustaining us, in the favorable sentiments we have expressed in relation to him.

As far as we are at prosent informed on the subject, he has taken the lead in one of the boldest projects that the votaries of *physical science* ever *have* instituted, or ever *can*—an attempt to solve at once the most recondite and momentous problem in the economy of nature. Our allusion is to the effort he has made, in the work we are examining to prove CALORIC to be what logicians term the VERA CAUSA—the *prime* and *immediate instrument* employed by the Deity, if not in the *production*, at least in the *original arrangement* and subsequent *government* of the material universe. To adopt the Doctor's own language, the more certainly and definitely to express his meaning. He has first virtually represented caloric as the only *self-moving material* substance in creation, every other sort of matter being held *motionless* either by *its own nature*, or by something tantamount to a *vis inertiæ.* Resting on this position as *a fact*, he has next attempted to prove his *self-moving* article to be the efficient and immediate source of all the "*mechanical, chemical, and vital agencies in the phenomena of nature.*" In other and perhaps more explicit and definite terms, to be the MATERIAL VICEGERANT of the Deity, in every movement that occurs in the UNIVERSE OF MATTER—whether that matter be *organized and living*, or *unorganized and dead.*

Such is our author's theory. And whether it is the pure product of reason and philosophy, or, to some extent, the creature of fancy and fiction, we may perhaps hereafter hazard an opinion. At present however we shall only say (but we say it *confidently*) that the fearless effort of the writer, which critics of a colder temperament and more cautious disposition may probably pronounce extravagant and unsafe (if not still *more condemnable*) is, in our estimation, indicative of an exceedingly rare expansion of mind and loftiness of purpose, boldness of enterprise, and sublimity of thought. And it need hardly be even surmised, much less maintainted by argument, that, without such mental attributes, success in *new and arduous branches of knowledge* can never be achieved. For, to such achievement, ambition and enthusiasm, intrepidty and firmness of purpose are no less essential than they are to the attainment of success in any other form of enterprise, whether in civil or military life. And though we do not contend that Dr. Metcalfe has been successful in the execution of his entire scheme of inquiry, yet does truth compel us to say, that he has thrown more light on many parts of it, and treated the whole of it with more ability, than any other author, whose writings we have examined. In no ordinary degree has he thus benefitted science by his masterly effort. Nor does the benefit conferred on it by him consist alone in what he has himself performed. He has presented to the ambitious an example that will be followed. He stands therefore in the attitude, and character of a public teacher, the most valuable portion of whose instruction to the community consists in his inducing others to instruct themselves, infusing into them a spirit of emulation, and communicating to them judicious directions to that effect.

Other philosophical naturalists, therefore, awakened to action by the example of Dr. Metcalfe, will not fail to engage in the inquiry, in which he has so ably and creditably preceded them. And, whether they concur with him, or oppose him in opinion, the issue will be the same. Further light will be elicited, by their labor, and science and the interests of mankind will be subserved.

Another trait which adds not a little to the extraordinariness of the production before us is the depth of its erudition, and the immense amount of knowledge it embodies in so moderate a compass. It ranks with the most erudite works we have ever perused. Nor is the *derivative* knowledge it contains of a common order, or extracted from common sources. On the contrary, it is itself of the highest and choicest kind, and is drawn from the most substantial authorities. It is not groundless eulogy, but sober truth, to say of it, that not a little of it has descended to us from the ablest and most highly and justly accredited authors of every civilized and enlightened country, and of every age, from the earliest era of the history of science and letters, to the present period.

It commences, as far as they are known, with the sentiments of men most noted for knowledge and wisdom among the Egyptians, Chaldeans, and primitive Jews, passes to the sages and philosophers of Greece and Rome, when they were the guiding lights of the world, and pays a due and not unprofitable regard to the few scattered and twinkling stars in the science of the Dark Ages. Commencing again with the revival of letters, the author makes deeper and more valuable draughts on those who from age to age, have written on the subject of his own researches. And it is especially at this period, in the progress of science, that his selections of knowledge begin and continue to be made with great judgment, and applied to the object he has in view with a corresponding degree of aptitude and force. He has here shown himself to be thoroughly acquainted with the writings, and their bearing on the subject of his inquiry, of Paracelsus, Van Helmont, and Stahl, comprehending as well the wild and evenscent notions, as the sound and lasting philosophy of those illustrious men. Nor is he less familiar with the works, their applications and value, of Copernicus, Galileo, Kepler, Newton, Laplace, and the Herschels in astronomy—with those on chemistry by Black, Lavoisier and Laplace, Priestley, Davy, Berzelius, Liebig, and Farraday—with those on geology by Cuvier, Lyell, and the geologists generally of Great Britain and France—with those on physiology, by Muller, Tiedemann, Magendie, and Carpenter—with those of Homer, Pindar, Virgil, Shakspeare, and Milton, in poetry—with those on mental philosophy by Locke, Brown, Gall, Spurzheim, Combe, and phrenologists in general—with those of Bacon, which embrace much of the philosophy of every department of nature—and with those of scores on scores of other authors (all of them distinguished) whose names a want of time and room forbids us to recite. And singular as it may appear, notwithstanding the immense variety, and the difference from each other, of the subjects on which those several authors have written, and the different modes in which they have treated them, Dr. Metcalfe has extracted from each of them matter illustrative of his theory of caloric, and, as he believes and ably contends, in confirmation of its truth. Such is the plastic and assimilating power of his mind, that he derives for his theory nutriment from every department of science and letters—the writings of the apostles and prophets not excepted.

This leads us to give another very striking representation of the work we are considering. It may be regarded, much more than any other production of the sort and size we now recollect, as *an epitome of an entire library of a very elevated and rare description*—of such a library indeed as does not, we apprehend, exist in the United States. Yet, so ample are the resources, and so dextrous the management of our author, that he has reduced it, vastly diversified as it is, to what may be called (if the phrase be admissible) a *multiplex unity;* by making it all bear directly or indirectly on a single subject—*his belief in the universal agency of caloric.*

Notwithstanding, however, the large amount and manifold sorts of *derivative matter*, which our author has embodied in his work, it is not to be inferred that he has not also manifested in it much independence and *originality* of mind. Far otherwise.

It need hardly be remarked, that originality, in the examination and discussion of subjects, is of two kinds. Of these, one consists in the discovery and employment of *new facts;* and the other in the placing of *new constructions* on facts already known—or the drawing from them of *new inferences*, by means of new associations and arrangements of them.

Of his possession, to an unusual extent, of the *latter* of these forms of originality, Dr. Metcalfe has exhibited in his volumes an abundance of testimony. And we need not remark, that, in doing so, he has shown himself to be endowed

3

with a much higher order of talents, than he would have done, by merely giving evidence of his possession alone of the *former*. By industry, labor, and steady perseverance, a *common mind may discover and amass facts*; but nothing inferior to the mind of a *philosopher* can analyse, interpret, and classify them, and apply them to the high and important purposes, for which they may be fitted. To the simplest observer *steam* has been long known to exist, and to possess no inconsiderable power of action. But, it required the genius of a Watt or a Fulton to perceive the numerous and invaluable uses which it now subserves, and to devise and direct the means for the proper application and employment of it. School-boys had, no doubt for centuries, been in the practice of amusing themselves by the raising of kites; but it belonged to a Franklin to employ that simple piece of machinery in disarming the thunder-cloud. And instances to the same effect might be indefinitely adduced. Nor are we able to call to mind any writer who is richer in the knowledge of them, or more dextrous in the application and use of them, than Dr. Metcalfe. By such dexterity he often illustrates and strengthens momentous opinions by facts known to every one, and usually regarded as trivial and insignificant. Of this the following is an instance, which, whether conclusive or not, bespeaks a mind abounding at once in ingenuity and resources.

The Doctor contends that caloric is the immediate and efficient cause of every form of attraction—centripetal, cohesive, chemical, and vital. And he thus applies it to account for that of cohesion.

Between the particles of *dry* pulverized earthy matter, whether silicious, aluminous, calcarious, or of any orher description, there exists no attraction. Hence dry weather and dusty roads are inseparable concomitants. But a fall of rain or a dash of water in any other way produces in the condition of things an entire change. Immediately the attraction of cohesion binds together the *wetted mass;* and, instead of continuing dusty, the roads become muddy. In other words, their surface becomes adhesive and tough. And, without expounding his scheme of reasoning on the subject, we shall simply observe, that, in perfect conformity to his general doctrine, and with no little address and ingenuity, Dr. Metcalfe attributes the change to the action of caloric. And, on the same principle does he explain the well known *fact*, that *hot water* penetrates and softens substances, and dissolves all such as are soluble in it, much more speedily and completely, and also in larger quantities than *cold* water. The solution is attributable to the attraction of caloric for the substance dissolved.

There is attached to Dr. Metcalfe's work another peculiarity, which adds not a little to the extraordinariness of its character, and the interest it is calculated to excite, especially in the West. And not in the West alone, but throughout the whole of the United States. We allude to the history of its authorship, which, in some of its elements, is all but romantic.

Dr. Metcalfe is a native of Kentucky, where he was born and received the rudiments of his education, before that State had completely shuffled off the sobriquet of 'Back-woods;' when schools of learning there were but little else than a name; and when, in the entire region, there existed nothing that could be called a library, and but few readers of such books as could be found—when in fact the buffalo, the bear, and the Indian had not yet entirely retreated before the hardy hunters of the frontier.

Notwithstanding this want of instruction, example, and other incentives to awaken it, our author exhibited, from his boyhood, a taste for letters. In testimony of this, and as the fruit of it, he wrote and published, when but a youth, a sketch of the life and adventures of that prince of pioneers and rangers, Daniel Boone. And though that production manifests something of the *germ of scholarship* and *Belles Lettres*, yet may it be stated with sufficient correctness, that nearly the whole of those accomplishments, as a writer, for which he is now so conspicuous, is the product exclusively of his subsequent labors.

The Doctor, as the title-page of his volumes shows, was graduated in medicine in the Medical Department of Transylvania University. He received his degree in that institution, in the spring of 1822 or 1823, and soon afterward removed to the State (*Territory* we believe at the time) of Mississippi, where he married, and, for several years, pursued industriously and successfully a professional life. But he was never, as we have been informed, content with his situation and prospects in that place, chiefly because his opportunity for the cultivation of the branch of science, in which he most delighted, was very limited and unpromising. Accordingly, on the death of his wife, which occurred a few years after his marriage, he abandoned his establishment in the south, and removed to the city of New York, in the hope of being able there to prosecute his favorite study, with the aid of all requisite advantages, to the completion of his design. But he found in a short time that he was mistaken, the libraries of that city being insufficient for his purpose, on account of not containing many of the works which it was necessary for him to consult. Having remained there, for a few years, and published a pamphlet on Caloric disclosing the nucleus of his views respecting its agencies in the economy of nature, he sailed for England, we believe, in the year 1835, to continue in London, aided by the libraries and other resources of that metropolis, his inquiries into the same subject.

As matters eventuated, that may be regarded as the boldest movement of his life, and the most striking feature in the history of his researches. Without the slightest indulgence in a spirit of exaggeration or undue compliment to our author, it may be compared to the course adopted and pursued, in quest of knowledge, by Pythagoras, Thales, Plato, Hippocrates, and other philosophers and sages of Greece; when they visited for that purpose Egypt, and other enlightened portions of the East, and long remained in them for the completion of their designs. Like them Dr. Metcalfe visited foreign countries, because they opened to him sources of knowledge far superior to those he could have access to at home, and actually remained there a *self-expatriated* sojourner, for the protracted period of *ten years.* Nor did he thus virtually exile himself for the enjoyment of what the world calls pleasure and amusement—except that he converted into sources of such enjoyment, his *researches and studies*, which, to ninety-nine persons out of every hundred, would have been *serious* and very *unwelcome labor.* He experienced, moreover, during his long residence abroad, inconveniences and evils other than those of mere expatriation. It is no *disparagement* to him, but the *reverse*, to say (and it may be truly and emphatically said) that he perseveringly adhered to his resolution and labors, amidst occasional pecuniary embarrassments and wants, to which, without incurring degradation, the concomitant of irresoluteness and pusillanimity, or the slightest charge of a deficiency of manliness, the stoutest and hardiest spirit might have yielded. But he determinately met and vanquished all the difficulties that lay in his path, finished his task, and returned to his native country, bearing on his brows, in no ordinary degree both rich and rare, the bays of victory, bestowed by foreigners. For foreigners have not withheld from the product of his labors an abundant tribute of approbation and praise. Much more therefore should that production receive, from the justice of his countrymen, the reward to which it is so eminently entitled.

Such then is the work of Dr. Metcalfe, hastily and somewhat desultorily described—and such a few of the reasons why we have pronounced it an "extraordinary production." And we cannot here refrain from repeating, what we have already less confidently intimated, that none but the high qualities of mind and spirit, which our author exhibited, during the entire period of his scientific enterprise, could have borne him successfully and triumphantly through it. We wish it however to be distinctly understood, that by the term "*triumphantly*" we do not mean to express our entire concurrence with the Doctor, in all his opinions. Our design is only to record by it our admiration of the mental ability and heroic spirit displayed by him, in the midst of sacrifices, difficulties, and privations, with which nothing but an instinctive and confident anticipation of success could have induced him so long and laboriously to contend. For it may be reduced to an axiom, that no person *will* so contend, except, as the result of a deliberate examination of his powers and qualifications, he feels in himself a fitness to succeed.—But, in justice to Dr. Metcalfe, as well as to our readers, we must now permit his volumes to speak for themselves. And this we shall do, by first stating the point or points which the Doctor proposes to prove, and then giving, in his own words, his matter of proof.

He commences his work by an interesting representation of the views of certain distinguished philosophers of antiquity, respecting the nature of *elementary fire*, which was no doubt identical with our *modern caloric.* He alleges that, as evidenced by the terms employed by them, in expressing their conceptions of it, they regarded it as a substance of an etherial and highly sublimated and refined, if not of an actually *spiritual nature.* Of course they must have attributed to it great power and efficiency of action. But he does not tell us precisely what this action was.

His next step is an attempt to prove, in opposition to the hypothesis of Lord Bacon, Sir H. Davy, Count Rumford, and others, that caloric, instead of consisting in *mere motion*, is a distinct substantive form of imponderable matter, possessing, as already stated, powers of action next in order and standing to Omnipotence itself. In his attempt to establish the *materiality* of caloric, and its independence of every other kind of matter, we think him as successful as *facts* can render him. A summary of his leading arguments is as follows:

"1. That it may be added to and subtracted from other bodies, and measured with mathematical precision, as all good thermometers demonstrate:—

"2. That it augments the volume of bodies, which are again reduced in size by its abstraction:—

"3. That it modifies the forms, properties, and conditions of all other bodies, in an endless variety of ways:—

"4. That it passes by radiation through the most perfect vacuum that can be formed by means of the air pump, in which it produces the same effects on the thermometer as in atmosphere:—

"5. That it exerts mechanical and chemical forces which nothing can restrain, as in volcanos, the explosion of gunpowder and other fulminating compounds:—

"6. That it operates in a sensible manner on the nervous system, producing intense pain, and disorganization of the tissues when in excess.

"But if caloric were a mere property or quality, how could it be taken from one body and added to another? Or if it augment the volume of other bodies, must it not itself have volume, occupy space, and therefore be a material agent? Would it not be mere jargon to speak of the radiation, reflexion, convection, and conduction of a mere quality, or immaterial property? And if caloric were only the effect of vibratory motion among the particles of ponderable matter, how could it radiate from hot bodies without the simultaneous transition of the vibrating particles? But it is certain that when iron, copper, and other metals are heated to any temperature below the point of ignition; like boiling water, they give off caloric freely, without any sensible loss of ponderable matter."

Another extract containing a breviat of Dr. Metcalfe's views of the power and general agency of caloric in the material universe, we recommend to the reader, as worthy of all the attention and reflection he can bestow on it. And, if he be not prepared or inclined to comply with our recommendation, our next advice to him is, not to read it at all. A *common-place* perusal of it will be useless.

"Having thus proved that caloric is a material agent, I now proceed to show that it is a self-active principle, capable of moving itself, and of generating motion in all other bodies. The cardinal facts which connect its agency with the general theory of physics, may be reduced to the followin propositions:

"1. That the activity or moving power of all bodies, is directly in proportion to the amount of caloric around their particles:—

"2. That all molecular motions, whether centrifugal or centripetal, may be resolved into the law by which caloric repels its own particles, and attracts those of ponderable matter, with forces that vary inversely as the squares of the distance:—

"3. That the quantity of motion in the world, whether mechanical, chemical, or vital, is in proportion to the mean temperature of different latitudes, ceteris paribus, and diminishes from the equator to the poles:—

"4. That the centrifugal force by which planets are impelled through their orbits, is directly in proportion to the heating power of the sun; and like gravitation, is inversely as the squares of the distance:—

"5. That the aggregate vital energy of animals, and the development of their organization, are exactly in proportion to the amount of caloric obtained by respiration, and combined with their tissues:—

"6. That every variety of electricity is convertible into caloric, and the latter again into electricity; consequently, that they are modifications of one and same principle:—

"7. That the directive power of the compass needle diminishes from the isothermal equator to the points of lowest mean temperature, which are the magnetic poles; and that all its variations correspond with the variations of terrestrial temperature:—

"8. That caloric is the active principle in light, whether radiated from the sun, or generated by ordinary combustion, friction, percussion, phosphorescence, or the electric discharge.

"That caloric is a self-active principle, might naturally be inferred from the fact, that every change in the temperature of bodies is attended with motion among their particles. It has also the power of moving from one place to another, and even through the vacuum of an air pump, without any projectile impulse from the particles of ponderable matter; as when it radiates from hot bodies. And that it has the faculty of generating motion among the particles of other matter, would appear from all the phenomena of nature with which we are best acquainted.

"For example, under the ordinary pressure of the atmosphere, and at, or a little below the temperature of 32°, the particles of water exist in the solid and apparently fixed state. But if 140° of caloric be added, their mobility is so far increased that they glide freely over one another, and assume the fluid form. If more be added, their activity is still further augmented by every degree of temperature, up to the boiling point, when the whole is found to be in a state of rapid intestine motion. And if at this stage of the experiment, the water be kept over a furnace, until it has received about 1000° more of caloric, the boiling liquid is converted into steam, the moving force of which is exalted by every addition of this active principle, which has the power of converting all other bodies from solids into liquids, vapors, gases, and flame, or luminous incandescent particles."

Were we about to invite the truly philosophical mind to an intellectual banquet of the highest order, we could not perhaps better accomplish our purposes, than by recommending to it a careful perusal—not to say *study* of every paragraph, from page 12 to the close of this chapter, which ends at the bottom of page 46. Although the pages referred to are interspersed with a few sentiments, in whose correctness we cannot concur—that is a consideration to which, at the present moment, we attach no weight. We are now speaking of *manner* and *character*, rather than of matter. And so able is the discussion involved in our recommendation, that it cannot fail to command the admiration of every enlightened and unprejudiced judge, by whom it may be examined.

The next topic, to which we shall specially invite the attention of the reader, is our author's attempt to prove the identity of caloric and atmospheric electricity. To the discussion of that point he has devoted an entire chapter (the *first* of his *third* book) and it is among the most able and interesting that his work contains. Still however is it here and there dotted with a few obscure spots (*rari nantes in gurgite vasto*) which, though *analogy* may sustain them, require a stronger and clearer light, united to the *direct* influence of additional *facts*, to identify them completely with truth and science.

It would be exceedingly gratifying to us to present to our readers an epitome of this chapter, were the end attainable. But it is not; because the chapter is itself an epitome of a large mass of matter, which cannot, as it appears to us, by either compression or abstraction, be any further condensed.

To communicate to our readers therefore some knowledge of the character of the chapter, the most suitable expedient we can adopt will be, to lay before them a few extracts bearing more or less directly on its leading purport—to show the *simplicity* at least, if not the *unity* of the source of action, in the seeming *multiplicity* and *complexity* of the economy of nature. The following extract will illustrate our meaning:

"The more profoundly we scrutinize her operations, the more we discover of simplicity and unity of power amidst all her diversified movements and transformations. Who could have suspected without a general survey of the widely extended provinces of nature, that the aggregation of liquids into spherical drops, and their adhesion to solids, are resolvable into the same power that determines the solidity of and rotundity of the earth?—which causes rain to descend from on high, rivers to flow towards the sea, and planets to revolve around the sun?—yet these various movements were traced by Newton to one and the same law.

"What can be more unlike to the mass of mankind, than the opposite forces of attraction and repulsion, contraction and expansion? Yet I have proved that they are both produced by one and the same agent; which in certain proportions binds the atoms of ponderable matter together, as in cohesion and chemical union; while in other proportions it diminishes or destroys their cohesion, as in liquefaction, vaporization, combustion, &c., by which the forms, properties, and powers of all bodies are perpetually changed."

"If, then, caloric be a universal principle of action in nature, and immediately connected with all the phenomena of motion, what is electricity? Is it a distinct fluid, *sui generis?* or is it a modification of the igneous principle? These questions, so intimately connected with the whole theory of physics, have never yet been satisfactorily answered. If electricity were the generic moving power of nature, it ought to be every where present. But so far is this from being the fact, that it is only under peculiar circumstances that it is developed so as to be appreciable by the senses; whereas, it is impossible to realize the absence of heat, which is indispensable to all the phenomena of climate, season, the growth of vegetation, and the life of nature."

In the course of his remarks on the subject, our author shows (what nobody indeed denies or doubts) that caloric is the immediate cause of evaporation; because, other things being alike, in proportion to the amount of *sensible caloric* (constituting *temperature*) in any place, is the amount of vapor produced—that wherever there is *evaporation*, *there* is also *electricity;* and, other things being equal, the quantity of the latter is in direct proportion to that of the former.

As clouds are the product of evaporation, and contain a large amount of latent caloric, the lightning which bursts from them is nothing but a stream or ball of condensed caloric in rapid motion.

In tropical climates there is more caloric, evaporation, and rain, than in temperate ones; and proportionally more of lightning and thunder.

In temperate climates caloric (temperature) evaporation, and lightning superabound alike in summer, and decline alike in autumn and winter. And, in polar climates, they are proportionately wanting throughout the year.

Caloric and lightning are identical in their action in melting metals, setting fire to combustibles, and producing flame.

During thunder-storms an abundant discharge of lightning is known to be, as a *general* if not an *unfailing* occurrence, followed by an abundant precipitation of rain. The cause is obvious. The lightning is a modification of the caloric which suspended the water of the cloud in a state of vapor; and, the suspending agent being suddenly discharged, in the form of lightning, the vapor it had produced and upheld is immediately condensed into water, and descends to the earth, in the form of rain. Our author's views on these points we shall submit to our readers in his own words.

"The cardinal facts which connect the phenomena of lightning with the theory of rain, may be reduced to the following propositions:

"1. In the torrid zone, where evaporation and rain are most copious, the amount of lightning is greatest. There is also far more during summer than winter in the middle latitudes, and scarcely any in the polar regions.

"2. In those parts of the world where there is no rain, there is no lightning, as at Lima in Peru; nor is there any in Egypt, Palestine, and other parts of the world during the prevalence of dry weather. It therefore follows,

"3. That where there is no condensation of aqueous vapor, there is no lightning.

"The most superficial observers of nature have been impressed with the immediate connexion between lightning, intense heat, and rapid precipitations of rain. By a careful analysis of the phenomena it becomes self-evident, that if caloric be the true and only cause of evaporation, its condensation and precipitation can be effected only by the evolution of the same agent.. During winter, the quantity and elastic force of vapor in the atmosphere are comparatively low in the middle latitudes, where it is condensed gradually on meeting with colder air. During spring, evaporation augments with increase of temperature, when masses of warm and cold air often meet, causing frequent showers of rain, and sometimes hail; still without much thunder and lightning. But during summer, when the temperature becomes tropical, and the atmosphere saturated with highly elastic vapor, we have tremendous explosions of thunder and lightning, with rapid precipitations of rain and hail. Corresponding with the temperature of the torrid zone, and the amount of evaporation, thunder storms occur almost daily during the rainy season, on the coasts of India and S. America."

We shall here extract a passage to show the accuracy and ability of Dr. Metcalfe, as a logician, no less than his soundness and depth as a philosopher. For, though he never deals in the technicalities of syllogism, there runs through his writings a closeness and strength of reason and argument, but rarely found in physical productions. His logic, when he resorts to it, is as solid and impenetrable, as that of Crawford in his celebrated treatise on the subject of phlogiston—or as that of the well-known logical treatise of Blane, the precise title of which we do not, at this moment, remember.

The passage we are about to quote contains Sir John Herschel's celebrated and well-founded rules for detecting the *common cause* of a large mass of unclassified facts, and our author's matter of proof that he has conformed to them, in his inquiry into the identity of electricity and caloric. The rules are as follows:

"1. Invariable connexion of the cause and effect, unless prevented by some counteracting cause.

"2. Invariable negation of the effect, with absence of the cause, unless some other cause be capable of producing the same effect.

"3. Increase or diminution of the effect, with the increased or diminished intensity of the cause in cases which admit of increase and diminution.

"4. Proportionality of the effect to its cause in all cases of *direct unimpeded* action.

"5. Reversal of the effect with that of the cause. He adds, that we are not to deny the existence of a cause in favor of which we have a unanimous agreement of strong analogies, though it may not be apparent how such a cause produces the effect.

"The above general rules apply with great force to the connexion between temperature and the phenomena of lightning.

"1. It has been shewn, in accordance with the first rule, that a certain range of solar *temperature* is indispensable to the existence of *lightning*, which are therefore invariably connected together as antecedent and consequence, or cause and effect.

"2. It has also been shewn, in conformity with the second rule, that in the absence of condensation and precipitation of vapor there is no lightning:

"3. That other things being equal, the amount of evaporation, condensation, and lightning, are increased and diminished with the augmentation and decrease of temperature:

"4. That the quantity and splendor of this wonderful meteor are proportional to the accumulation and intensity of solar heat, from the equator to the poles—and

"5. That the effect is *reversed* on passing from the regions of perpetual cold to the torrid zone, and from winter to summer.

"In reference to the 3d and 4th rules it may be observed, that the quantity of precipitation and lightning are greatly diminished, *cæteris paribus*, by *uniformity of temperature*, as in the tropical portions of the sea far from land, and other parts of the world where the winds blow long in one direction; whereas changes of temperature are indispensable to the condensation of atmospheric vapor, and to the evolution of its caloric in the concentrated form of lightning."

We shall here mention, as an insulated fact, that our author often embodies in his foot-notes some of the most curious and interesting statements, and valuable expositions that are contained in his work. As a brief specimen of them we extract the following, which, by a simple analysis, whose subject is more or less familiar to every one, demonstrates the vast agency which caloric holds, and the stupendous power it exerts in the economy of our globe.

"It has been estimated by geographers, that about thirty-three cubic miles of water are discharged daily into the sea by all the rivers of the earth. So that if this calculation be correct, or even an approximation to the truth; and if the same proportion of rain falls into the sea as on a given area of dry land, it follows that nearly 140 cubic miles of water must be carried into the atmosphere every twenty-four hours, and again precipitated in the form of rain and snow. Such are the vast forces which caloric is continually exerting in the phenomena of meteorology, without any reference to those violent commotions of the atmosphere termed hurricanes, tornadoes, and other less impetuous winds. Nor is it unworthy of passing notice; that as the quantity of water diffused through the atmosphere is in proportion to the heating power of the sun, the barometer stands higher, on an average, within the tropics than in the middle or polar latitudes; as proved by the observations of Erman, laid before the French Academy of Sciences,—after my chapter on the barometer had been printed. More water is also converted into steam during the heat of the day, and more steam is condensed into water during the coldness of night, which is the most convenient period for rain, as it does not then impede the labors of husbandry."

The only other portion of Dr. Metcalfe's volumes which we can notice at present, is that in which he treats of the "*Influence of climate and season on the physical, intellectual and moral character of the human race, as shown by the difference of stature, magnitude of the chest, configuration of the brain, complexion, &c., among the various nations of the earth;*" constituting one of the most interesting, instructive and practically important sections of his work. And though we do not concur with him, in every element of the doctrine he maintains on the subject, yet does truth compel us to say, that he has handled it with more ability, than any other author whose writings we have perused. Unquestionably he has collected and embodied in it a greater amount of appropriate and illustrative matter, than we have ever previously witnessed within so limited a compass. And he has ascribed the whole scheme of operation which his remarks embrace, directly or indirectly, to the agency of caloric. That substance is, in his opinion, second only to OMNIPOTENCE itself, in the creation of hot, temperate, and cold climates—and in supplying them respectively with fertilizing rains, bringing down on them blighting frost and snow, or in parching them with drought and entailing on them sterility. Caloric is also, if not the immediate, at least the remote agent in the formation, *geologically*, as well as *meteorologically*, of the difference between a productive and a sterile soil. It is likewise chiefly instrumental in the prodcution of the diversified localities of mountain, hill, plain, and valley. Climate, locality, and soil create and maintain the immense varieties of the animal and vegetable tribes that people the earth. And they again contribute in no small degree to the differences in the aspect, and constitution, condition and general character of MAN. And HE, by his powers and long continued industry in agriculture and other pursuits, changes materially some of the leading elements of *climate itself*, and of course its influence on living matter of every description—*himself* not excepted. The climate of the West and South of Europe is by no means what it was ten centuries ago, when it was overshadowed by forests, and covered only by the *native* productions of its uncultivated soil. We are taught to believe by records, whose correctness we have no reason to queston, that the climates of Italy, Germany, and Great Britain are far different now, from what they were at the commencement of the christian era. So, as we are assured, is the climate of Palestine and of the regions adjacent to it. And, though we have no historical records positively to instruct us respecting it, yet, for a man of intelligence who has reflected on the subject, to believe that the climate of China is not changed in two or three thousand years, by the various *influences* of near half a million of millions of resident human beings, and those of their concomitances and appurtenances, is absolutely impossible. Even, in the United States, the climate has undergone no very slight change, since the country was first visited by the Cavaliers and the Pilgrims. Nor is it easy to believe, that in the days of its power and glory, both intellectual and corporeal, even the land of the Nile and the Pharoahs experienced the same arid, adust, and sand-storm atmosphere and its concomitants that inflict on it the numerous evils of climate which it suffers at present. And all those mutations, whether for better or worse, our author, we repeat, imputes to corresponding mutations in the agencies of caloric. Some of the statistical results of climate, which he specifies, are memorable and momentous. His purpose in them is to prove the life-giving and life-sustaining agency of caloric. The following extract contains some of the principal facts which he adduces—and they are exceedingly significant, and favorable to some of the principles of his theory.

"But as all animals are nourished by plants, or by animals that have lived on plants, it is clear that the capacity of any country to support a numerous and wealthy population, must depend chiefly on climate. For example, throughout the whole of Asia and North America, above latitude 50°, the mean annual temperature is below 52°, and the growth of vegetation is arrested for ten or eleven months in the year, while in the coldest places (which are the foci of magnetic power), it falls 60° and 70° below 0° during winter. The consequence of which is, that in the vast regions of Siberia, embracing an area of 4,010,000 square miles, the number of inhabitants does not exceed 3,600,000 who, from the nature of the climate, are compelled to obtain a miserable subsistence by fishing and hunting.

"In the still more desolate regions of British and Russian America, on a territory of 3,650,000 square miles, the native population does not exceed 200,000, who live mostly in snow huts, clothed with skins, and nourished by animals, obtained chiefly from the sea. Without agriculture, commerce, manufactures, or the possibility of uniting together under regular forms of government, they are reduced to a state of complete barbarism; and, like the scattered tribes of northern Asia, are mere dwarfs in stature as in mind.

"And if we pass to the elevated plains of ancient Scythia, in central Asia, where the climate is colder than any part of the northern hemisphere in the same latitudes, where bleak northerly winds prevail for eight or nine months in the year, the growth of vegetation is always imperfect; and according to Balbi, on a territory of 4,250,000 square miles, the number of inhabitants does not exceed 30,000,000, who for the most part lead a wandering or nomadic life, and have never made any considerable progress in civilization, science, literature, and the arts, if we except the brutal art of war; so that if we include the whole of North America above latitude, 50°, and of Asia above 40°, the population is only about 33,800,000, on a territory of nearly 12,000,000 square miles.

"But in the warm and temperate latitudes of Asia, south of latitude 40°, where the growth of vegetation continues from six months, as in the north of China, to ten months, and even the whole year, in the southern portions of the continent, the population is about 400,000,000, on a territory of 6,450,000 square miles. In India, it is 140,000,000 on an area of 1,200,000 square miles; while in China alone, between latitude 20° and 40°, it is about 200,000,000, making an aggregate of 340,000,000 on a territory of 2,840,000 square miles. From which it may be shown by calculation that if the climate of northern Asia and America were equally favorable, they might support a population of more than 1,400,000,000 on a territory of 12,000,000 square miles; whereas it is only 33,800,000. And what is their rank in the scale of intellectual and moral enjoyment?

"If we pass to the warm and temperate latitudes of Europe, where the growth of vegetation continues from six to ten months in the year, and the inhabitants are seldom pinched with extreme cold, or exhausted by excessive heat, the population is about 170,000,000, on a territory of 1,425,000 square miles. In the United States, the climate and soil of which are equally genial and fruitful, there is a territory of 2,300,000 square miles, with a population of 17,000,000; which, should it go on doubling every thirty-six years, will be 150,000,000 in another century. And if the period of doubling be then fifty years, it will be 600,000,000 in another century. Nor can there be a reasonable doubt, that improvements in science, agriculture, the arts, and the adoption of a chiefly vegetable diet, will afford the means of supporting in comfort a much greater population to the square mile, than has ever yet existed in any part of the world. And should the colonies of Great Britain, now pouring into many of the best portions of the earth, continue to multiply as at present, the language, civilization, intelligence, wealth, and growing prosperity of the Anglo-Saxons must ultimately prevail in every quarter of the habitable globe."

That all other things being alike, the temperature of a warm or even of a *hot* climate is most favorable to the production and multiplication of every form of *living organized matter*, man himself included, is perhaps true. But that it is most favorable to the highest and most efficient development and constitution of man, is not true. To that a more temperate climate is better adapted. We are inclined to believe that the salubrious places, insular and continental, in and around the Mediterranean, especially Greece and Italy—to which may be added, in other latitudes, Ireland and England, have been more productive of human greatness, than any other parts of Europe. Our reference is to the frequent production of a high order of both mind and body—but peculiarly of the latter. But we *know* them to be equalled, and we believe *surpassed* in their fruitfulness, in that respect, by a large tract of the United States. We allude to the entire hill and mountain region throughout the Union, but more particularly in Virginia and North Carolina. We have long believed, and often asserted, that, be the immediate cause what it may, the finest specimen of the human race, *as a community*, we have ever visited, is found in the hill and mountain counties of *central* Virginia. We have also seen some rare samples of the excellence of all the higher qualities of man among the inhabitants of the hills and steeps of Vermont—though the winter climate of that region is very severe, and the summer heat, for a time, exceedingly intense.

It is not therefore so much the occasional *extremity* of the temperature of climate, as it is its *long continuance* that prevents, in the human system, that perfection which it might otherwise attain. So resilient and recuperative is the constitution of man, that, though somewhat unstrung and debilitated in this country, by the burning heats of June, July, and August, it regains its tone and strength, under the congenial and friendly temperature of the other nine months. Hence, notwithstanding the high and relaxing temperature of our summer atmosphere, man attains to as much perfection, in the United States, as in any other portion of the globe. And though Americans themselves have *sometimes doubted*, and the people of certain European countries positively *denied* this truth, facts have already occurred and are still daily accumulating, which will not fail to convince them in common of the palpableness of their mistake, and completely vindicate the reputation of the United States from the groundless and disparaging charge of being the birth-place of any sort or degree of human inferiority.

Another bold and startling opinion of Dr. Metcalfe is, that light is not an independent form of imponderable matter, and does not radiate in that character from the sun. But, that it is the product of various sorts—indeed of all sorts of ponderable matter *dissolved in caloric*, or at least thrown by that agent into a state of flame or incandescence. Yet, extraordinary and improbable as, on first thought, that opinion will be generally held, it is strongly supported by multitudes of facts. Of these facts one class is, that, when in a state of combustion, different substances produce light of different colors—white, blue, red, green, yellow, and violet, as may be easily demonstrated by well-known experiments.—Nor can a doubt be entertained, that each color depends on some peculiarity in the combustible which emits it. Nearly the whole of many sorts of matter moreover, when consumed by combustion, is dissipated in the form of light. The light of flame is nothing but combustible and, of course, ponderable matter, volatilized and powerfully acted on by caloric. But, that our author's opinion may be fairly judged of, we shall, in justice to him, adduce his reasons for it, in his own words.

"That every description of ponderable matter is actually convertible into light by a sufficiently intense heat, or by electricity, will appear from the following undeniable facts:

"1. That the quantity of light generated by ordinary combustion, friction, or percussion, is always in proportion to the rapidity with which ponderable matter is ignited and volatilized.

"2. That the color of light thus produced always depends on the species of matter employed.

"3. That the electric spark (like that produced by the collision of flint and steel) consists of exceedingly minute portions of ponderable matter in a state of incandescence, as will be shown hereafter by the decisive and beautiful experiments of Fusinieri.

"4. That when the electric fluid is transmitted through the vacuum of an air pump, little or no light is produced, as proved by the experiments of De Luc, and afterwards by those of Sir H. Davy.

"5. That the most intense heat of a voltaic battery never produces any light, except when acting on ponderable matter; consequently, that light and heat are not identical, as maintained by some modern theorists; and that neither of them is generated by the mere vibrations of an æthereal medium."

The following portion of one of our author's foot-notes, we deem too interesting and important to be omitted in our reference to this topic.

"That caloric is a constituent of light, (as it is of all other bodies,) and is the active principle in its generation, is evident from the fact, that the most refractory gems and metals are transformed into luminous incandescent particles by a sufficiently intense heat, definite measures of which are required to produce the effect on different species of matter. It has also been proved by the experiments of Hulme, Dessaignes, Macartney, and others, that every species of phosphorescence is promoted (for a time) by moderate heat, and always extinguished by the coldness of freezing mixtures; while others have found that the light of luminiferous insects is much greater in vessels of oxygen gas than in common air. But that caloric is a distinct essence, and may exist independent of light, is manifest from the fact, that it converts solids into liquids, vapors, and gases, in the midst of perfect darkness; while it radiates from the earth during night, as it does from boiling liquids and other hot bodies, without being attended with any light."

In confirmation of part of this note, we shall add, that we have repeatedly read common sized and even small print, at night, by the light of half a dozen of large West India fire-flies, placed in a vial filled with oxygen gas. When placed in a vial filled with atmospheric air, those insects are much less luminous—though still much more luminous than the common fire-fly of the United States.

But although we have hardly yet commenced what can be correctly called a review of Dr. Metcalfe's volumes, we must notwithstanding hasten to bring our article to a close. To prepare, *in any compass* of letter-press, a competent review of such a work, is a task second in difficuly only to that of composing the work itself. And to perform such a task, in the compass of twenty, thirty, or forty pages, would be even more difficult, in some respects, than the composition of the original production. The reason is plain. To review satisfactorily is to judge and analyze severely the published thoughts and purposes of the author whose writings are reviewed, and to draw from them and commit to paper, in a generalized and compressed, but perfectly lucid and intelligible form, the characteristic substance of the matter they involve. And no one who has had the least experience in it can be ignorant that the process of generalization and compression of intellectual matter is far the most arduous labor a writer can encounter. A fitness for its performance requires at once a degree of concentrativeness and a power of analysis, which few minds possess.

As our author's volumes however cover two branches, distinct from each other—the *phenomena* and *operations* of caloric in the department of *inorganic* and *dead*—and the same in that of *organized* and *living* matter, we at first indulged the hope of being able to finish, in the present article, all we might hold it requisite to say respecting the former branch. And, should it be deemed expedient to introduce the subject again to the readers of this Journal, it was our design, in that case, in a subsequent article, to treat exclusively of the latter branch, which occupies the fourth, fifth, and sixth books, constituting more than half of the work before us, and will be generally regarded as the most interesting and important portion of it.

We regret, however, to find ourselves entirely disappointed in our hopes respecting the branch of our subject, which relates to the agency of caloric in the operations of inorganic matter. So spacious is that field, and so abundant and diversified the matter it contains, and the phenomena it exhibits, that we have been able to traverse and examine, in this article, but a moderate portion of it. That section of it moreover which is characterized by the most sublime displays of power and grandeur is nearly untouched by us. We allude to the magnificent representation which our author has given of the agency of caloric, in the production of earth-quakes and volcanic eruptions, with their concomitants and consequences, the upheaving or depression of large portions of continents, the creation and destruction of islands and mountains, and in its operations as the immediate source of the centripetal and centrifugal forces, to which we are indebted for the glorious pageant of the play in their orbits of not only the planets and satellites of the solar system, but of the entire host of heavenly bodies, through the whole scope of material creation. And superlatively extravagant or altogether groundless as that theory may perhaps be deemed by persons who have never studied, and therefore do not understand it (*ignorance* being *ten times* the cause of opposition to new doctrines where *knowledge* is *once*) Dr. Metcalfe has treated it so ably and brought to bear on it so many illustrative facts and analogies, as to render it not only worthy of the attention of enlightened philosophical inquirers, but a source of no ordinary degree of pleasure and instruction to them.

Not only does the perusal of the pages of our author's work, in which it is expounded, communicate knowledge itself; by the agreeable and peculiar exercise it affords, it excites, expands, and strengthens the mind, and throws it into a state of higher and more effective preparation for the reception of knowledge from other quarters.

We regret however the less, not having been able to give, in this article, a more comprehensive view of Dr. Metcalfe's work, as we are gratified to learn, and to be authorized to announce to the public, that an edition of it will be issued in a short time from the American press. And we hope the edition will be a large one, and would even counsel to that effect, that the countrymen of the author may be permitted to manifest, on an extensive scale, by an extensive purchase of it, their anxiety to peruse it, and the high estimation in which they hold it, in anticipation, *as an American production.* And we venture confidently to promise them, that no sooner shall they have made themselves familiar with its contents, than its *mere Americanism* will be thrown into shade, by its other higher and more valuable qualities.

One of the peculiar characteristics of our author, as a writer, is his never-ending solicitude to detect and comprehend the *causes* of things. The oft-quoted line of the poet,

"*Felix qui potuit rerum cognoscere causes,*"

seems never for a moment to escape from his memory. And by that recollection, and his constant endeavor to realize it in himself, he proves that he is actuated by the true spirit of philosophy. For so far as an inquirer applies himself to an investigation of causes and their connexion with effects, he acts, whether successfully or not, the part of a philosopher. And, in the work we are examining, Dr. Metcalfe has pursued that practice to an extent, to which we do not, at this moment, remember a parallel. So true is this, that he seems to have adopted, as a rule of self-government, never to incur the charge preferred by Lord Bacon against the philosophers of his time, and those who had preceded him, that, in their investigations, the most important facts connected with the fundamental constitution of nature had been overlooked by them; such as the "*cause of heat and light, weight and density, hardness and softness, solidity and fluidity, fomentation and putrefaction, germination and organization.*"

These are indeed facts and considerations of the utmost importance in the fundamental constitution of nature, with the causes of which philosophers should at least very strenuously endeavor to attain an acquaintance. And if they fail thus to 'endeavor,' they are so far wanting in the attributes of their calling, and incur the forfeiture of a corresponding portion of the proud title to which they aspire.

Very few persons however bestow on these matters the consideration they deserve. Almost every one regards them as trivial, common-place points unworthy of attention. Yet is the cause of the heat and light of a burning candle as full of interest, as much an instructive element of philosophy, as is the cause of the heat and light which emanate from the sun. Nor is a knowledge of the causes of weight, hardness, and softness less important than a knowledge of the causes of the de scent of ponderous matter, or of the centripetal and centrifugal forces which regulate the movements of the heavenly bodies. Indeed it might be easily shown to be, to a certain extent, more important, because more immediately connected with ourselves, and applicable to the common practical purposes of life. Yet is the latter knowledge highly esteemed and eagerly sought after, while the former is comparatively despised and neglected. So true is it that we seldom fail to concede to things rare and foreign an ill-judged and groundless preference to things common and domestic.

Very different, however, is the practice of Dr. Metcalfe. He sets a high estimate on the knowledge of *every cause*, and aims at the attainment of it; because it makes a part of the machinery of creation.

Finally; if the Doctor succeed in establishing the correctness of his theory of caloric, (and we do not oracularly assert that he will not) he will be one of the most illustrious benefactors of physical science (if not *himself the most illustrious*) that time has produced. His success, should he achieve it, will be the solution of the *gordian knot*, to effect which many of the master geniuses of every enlightened era and country have labored in vain. And whether he succeed completely or not, he will notwithstanding, by virtue of what he has already done, occupy a place among the most eminent writers of the age.

