# Erratum

**Published:** 1846-07

**Authors:** 


Erratum.—For *fo*mentation, read *fer*mentation, page 48, end of first paragraph.

